# Genome-wide identification and expression profiling of B3 transcription factor genes in *Populus alba × Populus glandulosa*

**DOI:** 10.3389/fpls.2023.1193065

**Published:** 2023-05-30

**Authors:** Mingke Wei, Hui Li, Qiao Wang, Rui Liu, Linxi Yang, Quanzi Li

**Affiliations:** ^1^ State Key Laboratory of Tree Genetics and Breeding, Chinese Academy of Forestry, Beijing, China; ^2^ Guangzhou Institute of Forestry and Landscape Architecture, Guangzhou, Guangdong, China; ^3^ College of Resources and Environment, Qingdao Agricultural University, Qingdao, Shandong, China

**Keywords:** B3, poplar, xylem development, co-expressed, lignin

## Abstract

B3-domain containing transcription factors (TFs) are well known to play important roles in various developmental processes, including embryogenesis, seed germination, etc. Characterizations and functional studies of the B3 TF superfamily in poplar are still limited, especially on their roles in wood formation. In this study, we conducted comprehensive bioinformatics and expression analysis of B3 TF genes in *Populus alba × Populus glandulosa*. A total of 160 B3 TF genes were identified in the genome of this hybrid poplar, and their chromosomal locations, syntenic relationships, gene structures, and promoter cis-acting elements were analyzed. Through domain structure and phylogenetic relationship analyses, these proteins were classified into four families LAV, RAV, ARF, and REM. Domain and conservation analyses revealed different gene numbers and different DNA-binding domains among families. Syntenic relationship analysis suggested that approximately 87% of the genes resulted from genome duplication (segmental or tandem), contributing to the expansion of the B3 family in *P. alba × P. glandulosa*. Phylogeny in seven species revealed the evolutionary relationship of B3 TF genes across different species. B3 domains among the eighteen proteins that were highly expressed in differentiating xylem had a high synteny, suggesting a common ancestor for these seven species. We performed co-expression analysis on the representative genes in two different ages of poplar, followed by pathways analysis. Among those genes co-expressed with four B3 genes, 14 were involved in lignin synthases and secondary cell walls biosynthesis, including *PagCOMT2*, *PagCAD1*, *PagCCR2*, *PagCAD1*, *PagCCoAOMT1*, *PagSND2*, and *PagNST1*. Our results provide valuable information for the B3 TF family in poplar and show the potential of B3 TF genes in engineering to improve wood properties.

## 1 Introduction

The plant-specific B3 superfamily constitutes one of the largest transcription factor (TF) families in plants. All members of the B3 superfamily contain an approximately 110 amino acid region called the B3 domain (Swaminathan et al., 2008). This domain is initially named because it is the third basic domain in the maize protein VIVIPAROUS1(VP1) (McCarty et al., 1991). The B3 domain of VP1 has a sequence-specific DNA binding activity (Suzuki et al., 1997). Subsequently, the B3 domain has been found in 118 genes of *Arabidopsis* and in 91 genes of rice. B3 TF genes are also present in green algae, mosses, liverworts, ferns, and gymnosperms (Marella et al., 2006).

The B3 superfamily encompasses several distinct gene families, including LAV (LEAFY COTYLEDON2 [LEC2]-ABSCISIC ACID INSENSITIVE3 [ABI3]-VAL) (Wang et al., 2012), ARF (AUXIN RESPONSE FACTOR) (Ulmasov et al., 1997), RAV (RELATED TO ABI3 and VP1) (Kagaya et al., 1999) and REM (REPRODUCTIVE MERISTEM) (Romanel et al., 2009). B3 TFs are involved in a variety of biological processes, such as seed development, embryonic development, etc., generally through polycomb silencing in plants. In *Arabidopsis*, the VAL subfamily genes *VAL1*/*HIGH-LEVEL EXPRESSION OF SUGAR-INDUCIBLE GENE 2* (*HSI2*), *VAL2/HSI2-Like 1* (*HSL1*), and *VAL3/HSI2-Like 2* (*HSL2*) are expressed in many organs throughout plant development. *val1 val2* double mutant exhibits embryonic callus formation on both roots and shoots, suggesting a role of *VAL1* and *VAL2* in repressing the embryonic developmental program. Germination is delayed in this double mutant, and the analysis shows that HSI2-dependent silencing of *DOG1* promotes the early release of seed dormancy (Chen et al., 2020). LAV family member LEAFY COTYLEDON 1 (LEC1), which is the Hap3 (HEMEACTIVATED PROTEIN 3) sub-unit of the CAAT-binding factor, controls various aspects of seed development from early embryogenesis to late seed maturation (Baumbusch et al., 2004). ABI3, LEC2, and FUSCA 3 (FUS3) also play key roles in the control of seed maturation in cooperation with LEC1 and abscisic acid (ABA) (Tsukagoshi et al., 2005). In *Vitis Vinifera L.*, overexpression of *VvFUS3* in tomatoes causes a reduction in total cell area and cell number, while the cell size of the fruit pericarp is increased. These results suggest that *VvFUS3* has a role in seed development by influencing the ABA signaling (Ahmad et al., 2022). ARF family member AtARF1 binds the upstream promoter regions of many auxin response genes (Ulmasov et al., 1997). Mutation of *ARF7* causes nearly an absence of lateral root formation, lacking of phototropism and auxin responses (Liscum & Briggs, 1995; Ruegger et al., 1997; Watahiki and Yamamoto, 1997). Both *ARF4* and *ARF3* regulate the abaxial-adaxial polarity in leaves and flowers in *Arabidopsis* (Pekker et al., 2005). RAV1 functions as a negative regulator of lateral root and rosette leaf development, and it is downregulated by the plant steroid hormone epibrassinolide (Hu et al., 2004). *GmRAV* negatively regulates short day (SD)-mediated flowering and hypocotyl elongation, and its overexpression in SD inhibits the growth of soybean leaves, roots, and stems (Zhao et al., 2008). The B3 gene also promotes flowering and functions in the maintenance of the vernalization response in *Arabidopsis.* For example, *FLC* (*FLOWERING LOCUS C*), a gene delays flowering, is transiently downregulated in the *vrn1*(*VERNALIZATION1*) mutant that has vernalization treatment, but levels of *FLC* RNA and protein are increased when the mutant is moved to normal temperature (Levy et al., 2002; Sung and Amasino, 2004). *Brassica oleracea BoREM1*, a putative ortholog of *AtREM34*, is specifically expressed in the cauliflower curd and involved in the determination of floral meristem identity (Franco-Zorrilla et al., 1999). In short, the B3 proteins of the LAV, RAV, ARF, and REM families are mainly involved in the signaling pathways of hormones including auxin, ABA, and brassinosteroid.

Wood formation is a specific biological process in trees. The major components of wood are lignin, cellulose, and hemicelluloses, which are deposited in the secondary cell walls (SCWs) of fiber cells and vessels in the xylem. These three polymers maintain the overall structure and strength of plant SCWs (Wessels et al., 2019). After years of efforts, the mechanism of lignification and SCW deposition has been preliminarily analyzed, and regulatory networks that regulate wood formation have been proposed in some review articles (Zheng-Hua and Zhong, 2015; Borthakur et al., 2022). The regulatory network for SCW thickening includes many NAC and MYB genes, such as NST1 to NST3/SND1 (Mitsuda et al., 2005; Zhong and Ye, 2006; Zhong et al., 2007), VND1 to VND7 (Kubo et al., 2005; Yamaguchi et al., 2008; Zhou et al., 2014), and PtrWND2B/6B (Zhong et al., 2011), are master regulators of xylem differentiation and SCW thickening. However, there are still more upstream regulatory genes for these master regulators, which need to be revealed. *VNI2* (*VND-INTERACTING2*) and *VND7* were co-expressed in the elongated ductal precursors of the root. Overexpression of *VNI2* significantly inhibited the differentiation of normal vessel cells. Transient transformation of the reporter gene showed that VNI2 is a transcriptional repressor, which can inhibit the expression of VND7-regulated vessel-specific genes. Sumoylation of LBD30 in poplar affected the expression of *SND1* and *NST1* in the transient transformation of protoplast systems. As LBD30 is sumoylated by SIZ1, this protein modification activates the regulatory network of SND1/NST1-mediated secondary cell wall formation in fibers. In addition, lignin is the most direct material for the study of wood formation. Lignin accounts for ~20% of SCWs, and it is polymerized by three hydroxy cinnamyl alcohols, including sinapyl alcohol, coniferyl alcohol, and *p*-coumaryl alcohol (Sarkanen and Hergert, 1971; Watahiki and Yamamoto, 1997). These alcohol precursors are termed the syringyl (S), guaiacyl (G), and hydroxyphenyl (H) lignin subunits, respectively. In angiosperms, lignin is polymerized primarily from S and G monolignols and trace amounts of the H monolignol (Wang et al., 2021). The biosynthesis of monolignols lignin occurs in several consecutive reactions involving 11 enzyme families and 24 metabolites, in a branched grid-like pathway (Vanholme et al., 2013; Wang et al., 2014; Sulis and Wang, 2020). These families include phenylalanine ammonia-lyase (PAL), cinnamate 4-hydroxylase (C4H), *p*-coumaroyl-CoA 3-hydroxylase (C3H), *p*-coumarate CoA ligase (4CL), hydroxycinnamoyl transferase (HCT), caffeoyl shikimate esterase (CSE), caffeoyl-CoA *O*-methyltransferase (CCoAOMT), cinnamoyl-CoA reductase (CCR), conifer aldehyde 5-hydroxylase (CAld5H, first named F5H, ferulate 5-hydroxylase), caffeic acid 3-*O*-methyltransferase (COMT) and cinnamyl alcohol dehydrogenase (CAD) (Zhao and Dixon, 2011). In this pathway, C3H catalyzes the hydroxylation of coumaric acid to form caffeic acid through the 3-carbon hydroxylation of aromatic rings of various phenol intermediates (Palafox-Carlos et al., 2014). The reduction of feruloyl-CoA to conifer aldehyde is mediated by CCR (Li et al., 2005). Coniferaldehyde is catalyzed by CAD to produce confieryl alcohol, G monolignols (Baucher et al., 1996). Coniferaldehyde can also be converted to sinapyl alcohol by CAld5H/F5H, COMT, and CAD, to produce S monolignols (Lapierre et al., 1999). Overexpression of *CAld5H* have a 64% increase in the S/G ratio in poplar, and co-transformation of *F5H* and *COMT* results in 2- to 3- times higher S/G ratio than *F5H* alone (Li et al., 2003). The lignin S/G ratio varies among different tree species, and it is a more important factor affecting the pulp yield than the lignin content (Del Río et al., 2005; Studer et al., 2011). High pulp yield is correlated with a high S/G ratio in wood biomass, so genes controlling S/G ratio in tree have great promising to breed pulp-specific trees.

Poplar is an ideal species to study the formation of wood, the important biomass energy material in nature. Although the pathway enzyme genes of cellulose, hemicellulose, and lignin biosynthesis and many upstream TF regulators have been identified, these studied regulators are only a small proportion of TFs. Among B3 TF family, PagVAL2-B1 is identified as an candidate regulator involved in wood formation, because yeast one hybridization shows that it can bind to *PagCAld5H2* promoter (Wang et al., 2021). Many remaining genes that are specifically expressed during wood formation may have potential functions in regulating specific wood properties. The involvement of B3 TF family genes in wood formation and their specific regulation on wood property formation needs investigation. In this study, we focused on the characterization of the B3 family in *P. alba × P. glandulosa*, and identified potential key genes involved in wood formation.

## 2 Materials and methods

### 2.1 Materials

Samples were collected from six-month-old trees that were grown in a greenhouse. After the bark was peeled, the xylem and phloem were scratched by single-end razors, and frozen immediately in liquid nitrogen. The tissues of leaf, xylem and phloem tissue from 10-year-old trees were from the stock in liquid nitrogen in a previous study (Li et al., 2021a).

### 2.2 Identification and annotation of B3 TF genes in *P. alba × P. glandulosa*

*P. alba × P. glandulosa* genome resource was downloaded from the Figshare database (https://figshare.com/articles/dataset/84K_genome_zip/12369209). To identify B3 family members, the HMM profile (PF02362) of the B3 DNA-binding domain from the Pfam database (http://pfam.sanger.ac.uk/) was used as input to perform an HMMER search in the *P. alba × P. glandulosa* genome with an E-value cut-off of 1e-3 following the HMMER guider (Horn et al., 2005). In addition, manual annotation is used to correct any inconsistencies between the predicted gene and its actual chromosome location.

All coding sequences (CDSs) were translated into amino acid sequences, and alignment of all B3-domain proteins from Arabidopsis and *P. alba × P. glandulosa* was conducted by MAFFT (L-INS-I algorithm) using the B3 domain amino acid sequences (Rozewicki et al., 2019). Subsequently, a phylogeny was generated using IQ-TREE Linux software and Model Finder (Chernomor et al., 2016).

### 2.3 The naming of B3 family genes

All identified B3 genes were named with consistent patterns, based on their subfamily association and phylogenetic relationships, as well as their subgenome locations (A, B). Each gene is named by starting with the abbreviation for the species name *Pag* (*P. alba × P. glandulosa*) followed by the name of the *Arabidopsis* gene with the most updated naming system from this subfamily (e.g. *VAL1* for *VAL-like genes*). The gene names including an A or B indicate the subgenome they are located in: for example, *PagVAL2-B1* and *PagVAL2-A1*. Putative alleles have identical gene names but with different subgenome identifiers (e.g. *PagVAL2-A1*, *PagVAL2-B1*). Genes belonging to one subgenome but at different chromosome locations were consecutively numbered (e.g. *PagVAL2-A1* and *PagVAL2-A2*).

### 2.4 Phylogenetic tree construction and location of B3 family genes in chromosome

The full length of protein sequences and conservation regions of all the identified B3 family members was aligned by MAFFT (E-INS-I algorithm). The alignments were trimmed using trimAl software, and then a phylogeny tree was inferred under maximum likelihood with IQ-TREE (Capella-Gutiérrez et al., 2009; Chernomor et al., 2016). The substitution model was determined using Model Finder, which is integrated into IQ-TREE. The best-fit model, JTT+F+G4, was selected based on the Bayesian information criterion (Capella-Gutiérrez et al., 2009). To evaluate the reliability of the phylogenetic estimate, Ultrafast bootstraps and a Shi-modaira-Hasegawa approximate likelihood ratio test were performed with 1000 replicates each. The phylogenetic tree was visualized on the iTOL web browser (https://itol.embl.de/) and Fig Tree V1.4.4 software.

Gene collinearity analysis and visualization within the B3 genome were conducted by TBtools (Chen et al., 2020). Detailed analysis methods are described in Li et al., 2022. The chromosome data and gene family file were mapped to their respective locus in the *P. alba × P. glandulosa* genome in the acicular diagram using shinyCircos in RStudio (Capella-Gutiérrez et al., 2009).

### 2.5 Analyses of motifs, domains, gene structures, and cis-acting elements B3 genes

The upstream (2 kb) regions of all identified B3 genes were extracted using the Fasta Extract software (Chen et al., 2020). Cis-acting elements were analyzed in promoter sequences through the PlantCARE online server (http://bioinformatics.psb.ugent.be/webtools/plantcare/html/). Conserved motifs of proteins were analyzed using MEME Suit 5.5.1 (http://meme-suite.org/tools/meme), searching up to 28 conserved motifs. Protein domains were identified using the Conserved Domain Database on NCBI (https://www.ncbi.nlm.nih.gov/Structure/cdd/cdd.shtml). The gff3 file provided by the genome was used to display the gene structure, including the exon, intron, and UTR regions using TBtools (Chen et al., 2020). The Gene Structure View module in TBtools was used to visualize the motif and domain of B3 genes.

### 2.6 Conservation region of the B3 proteins

A BLASTp was performed of all B3 proteins to *A. thaliana* with the threshold of 1e-5 to divide them into four families. The protein sequences in each family were firstly aligned by clustal in MAFF software; then the aligned fasta file was inputted into GeneDoc software (Nicholas and Nicholas, 1997) to show the conserved region of each type of B3 proteins. The conserved regions were then inputted into Jalview software (Waterhouse et al., 2009) to show the conserved sequences and seqlogo graphs of each type of B3 protein.

### 2.7 Collinearity and phylogeny analyses of B3 genes among different species

The multiple Chr layout, gene link, and gff3 files between two species in seven species were produced by the One Step MCScanX-Super Fast module in TBtools with the *E-*value of 1e-3, including *A. thaliana*, *P. trichocarpa*, *Gnetum montanum*, *Alsophila spinulosa*, *Physcomitrella patens*, *Spirogloea muscicola* and *P. alba × P. glandulosa*. The three files generated from two datasets were merged using the File Merge module of MCScan-X software, and homologous genes among different species were obtained from the merged genelink file. By extracting protein sequences of all these homologous genes, a phylogenetic tree among seven species was constructed using IQ-TREE by the maximum likelihood method with Ultrafast bootstraps (1000 replicates). The collinearity plots among different species were visualized using the Multiple Synteny Plot (MSP) module in TBtools software. The CDSs of B3 domain-containing proteins were identified from the genome and transcriptome databases of green algae, moss, fern, gymnosperm, and angiosperm (**Supplementary Table S1**) with BLASTP with the threshold of 1e-5 using B3s protein from *A. thaliana* as separate queries.

### 2.8 RNA-seq analysis

The expression patterns of target genes in three tissues, including xylem, phloem and leaf, were analyzed using the two RNA-seq data from ten-year-old trees (Li et al., 2021a) and six-month-old trees (Li et al., 2021b), respectively. Expression levels of 160 B3 genes and 86 midnight-blue module genes were analyzed by the DESeq2 software. The expression levels were quantified as log_2_ (transcripts per million) (log_2_tpm), and a heatmap was generated using MORPHEUS (http://software.broadinstitute.org/). The genes with undetectable expression levels were removed, and the remaining genes were clustered according to their expression correlation using Hierarchical (Metric: One minus Pearson correlation, Linkage method: Complete).

### 2.9 Reverse transcription-quantitative PCR

Total RNAs were extracted using the RNeasy Plant Mini Kit (QIAGEN, CA, USA), and reverse-transcribed to cDNA using a Prime Script TM RT Reagent Kit with gDNA Eraser (TaKaRa, Dalian, China). PCR was conducted using the Green Premix ExTagII (TaKaRa, Dalian, China) and detected by the Roche Light Cycler 480 II. Actin was used as the reference. The primers used are shown in **Supplementary Table S2**.

### 2.10 Gene co-expression networks and pathways analyses

By utilizing the WGCNA package in R, we were able to detect gene co-expression through analyzing the gene networks. The different model results were then used to visualize by Cytoscape. KEGG enrichment analysis was carried out to screen out the modules of genes related to wood formation. Among them, the midnight-blue enrichment module was selected for analysis. The correlation coefficient of genes in the network was calculated using the Pearson algorithm. The top eighteen genes with the highest weights were selected for drawing the co-expression networks. Eighty-six genes in the modules related to wood formation were used for co-expression analysis.

## 3 Results

### 3.1 Evolution relationship of B3 TFs and chromosome distribution of B3 genes *in P. alba × P. glandulosa*

**Figure 1:**
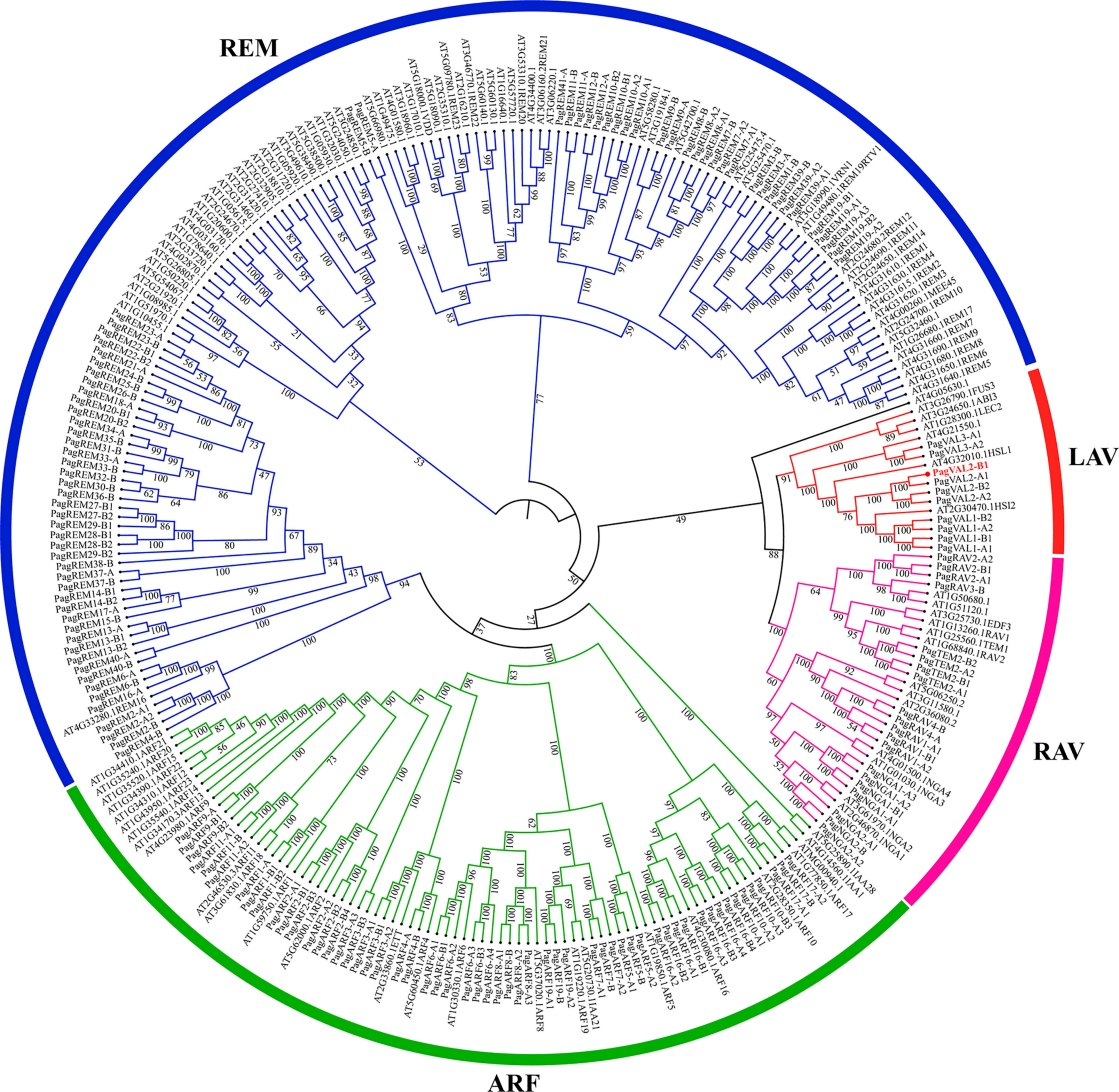
Phylogeny analysis of B3 family members. Colors represent different clades of B3 genes. The number on the tree branches represents the bootstrap value.

To identify B3 genes in the *P. alba × P. glandulosa* genome, we performed the HMM search using a Hidden Markov Model algorithm with the conserved B3 domain model (PF02362) as an inquiry. After protein sequences were trimmed based on the alignment, the protein sequences of 160 B3 proteins in *P. alba × P. glandulosa* were used to construct the phylogenetic tree, with B3 proteins from *Arabidopsis* (**Figure 1**).

These 160 B3 proteins were classified into four families, LAV, RAV, ARF, and REM, which contained 10, 20, 56, and 74 members, respectively. The LAV family in *Arabidopsis* consists of two subgroups, the LEC2-ABI3 subgroup and the VAL subgroup. However, the LAV family in *P. alba × P. glandulosa* only had one, the VAL subgroup. The gene number of RAV and ARF families in *P. alba × P. glandulosa* was similar to that in *Arabidopsis.* Among the four families, the REM family had the most varied gene numbers between *P. alba × P. glandulosa* and *Arabidopsis*. The results show that the LAV and REM families are more diverse than the other two families, which have constant gene numbers.

**Figure 2:**
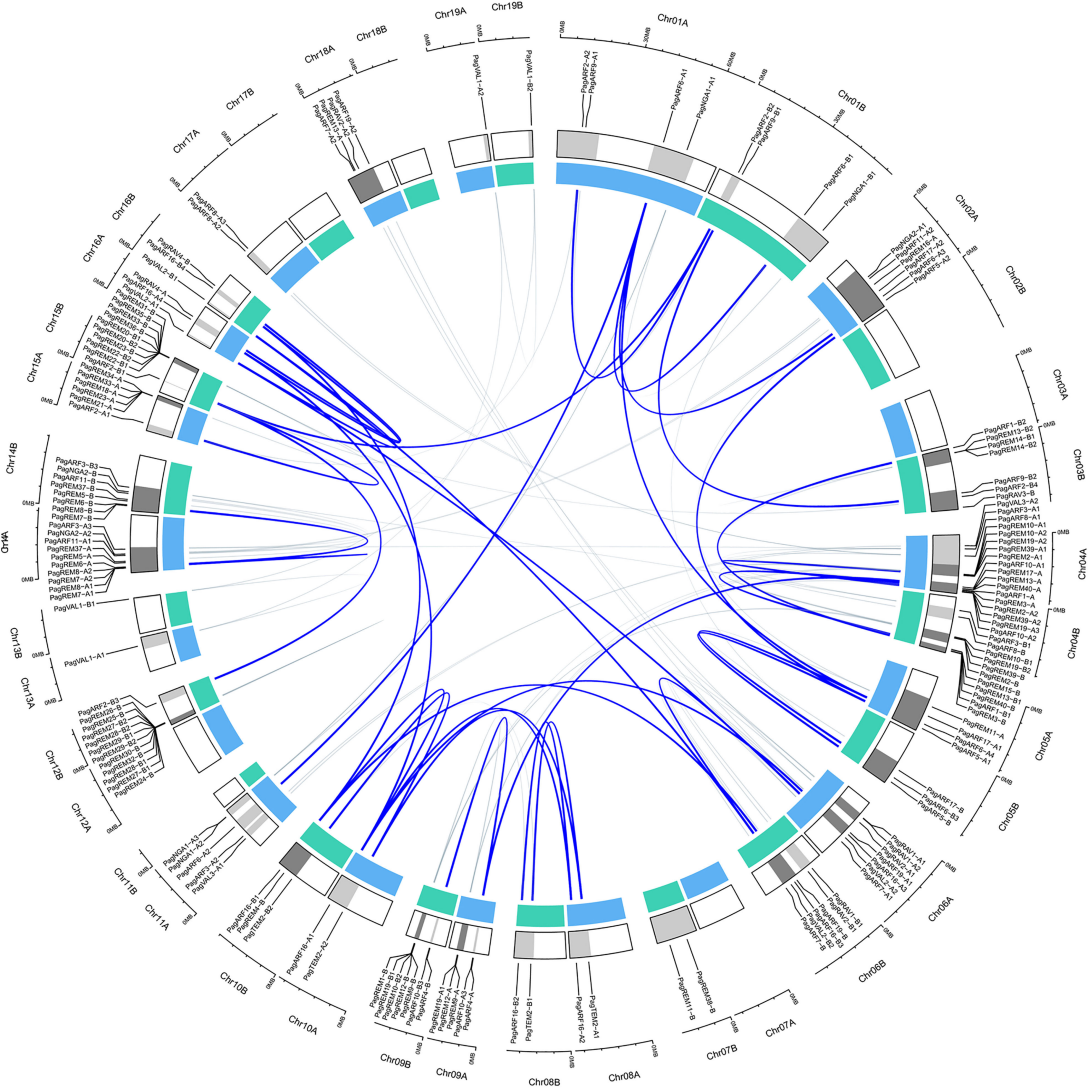
Distributions of B3 genes in the chromosomes of *P. alb × P. glandulosa*. The genes were mapped to their respective locus in chromosomes in a circular diagram using shinyCircos. Subgenomes are indicated by different shades of blue (inner track), and chromosomal segments are indicated by shades of gray (outer track). Homoeologous genes were inferred by phylogeny and linked with a grey line (inside of the circle). The highlighted blue line within the circus means the gene paralogous relationship of eighteen genes was highly expressed in the xylem.

We detected the distribution of the identified B3 genes in chromosomes. They were distributed unevenly on chromosomes (**Figure 2**). Chromosomes Chr04, Chr14, and Chr15 contained significantly more B3 genes than others did. The B3 genes in these three chromosomes were 29, 18, and 19, respectively, occupying 18.1%, 11.3%, and 11.8% of B3 genes on chromosomes. In contrast, no B3 gene was present in Chr02B, Chr07A, Chr12A, Chr17B, and Chr18B. There was no correlation between the length of the chromosome and the distribution of B3 genes on the chromosome.

### 3.2 Motifs, domains, gene structures, and promoter cis-acting elements analyses

**Figure 3:**
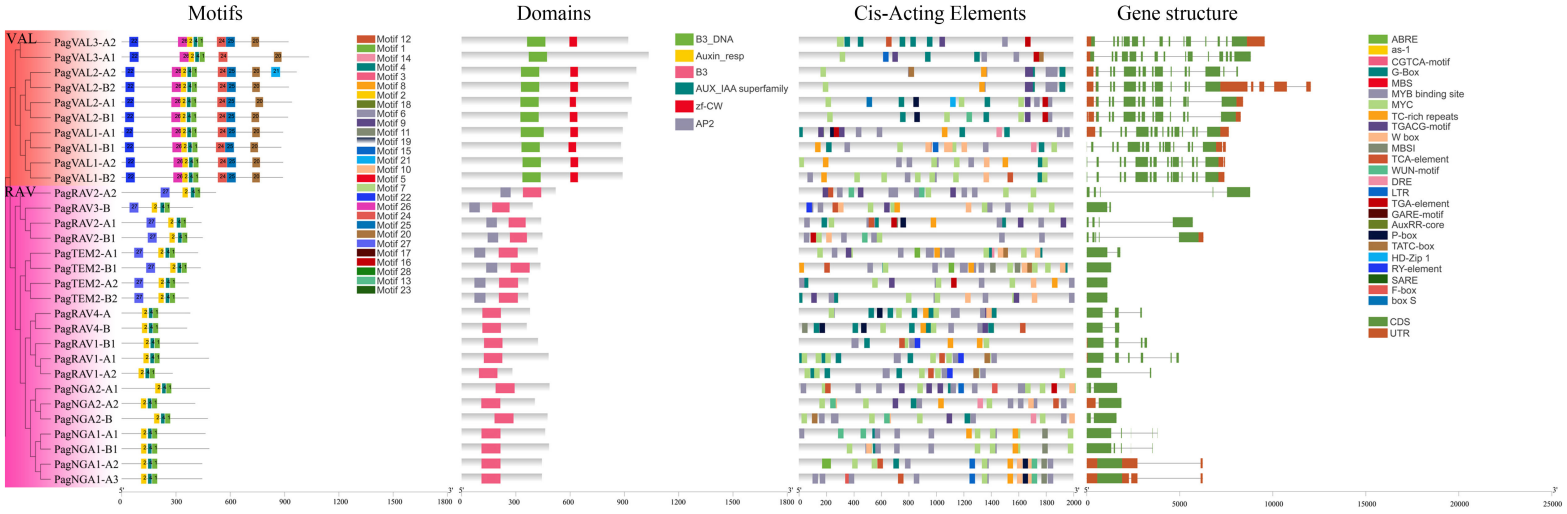
Motifs, domains, cis-acting elements, and gene structures of the LAV and RAV families. Different colors represent different motifs, domains, cis-acting elements, and gene structures. The ARF and REM results are in **Supplementary Figures S1**, **S2**.

**Figure 4:**
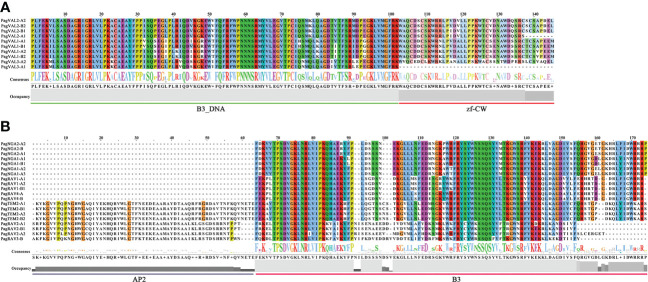
Sequence alignment and seqlogo of B3 gene families. **(A)** VAL **(B)** RAV. The same color in the column and the big size of the letters mean a highly conservative region. The ARF and REM domain sequence alignment are in **Supplementary Figures S4**, **S5**. The line underneath of sequences indicates residues of the domain consensus block.

To gain insight into the identified 160 B3 gene members, we performed domain, motif and gene structure analyses. The motif analysis showed that most B3 TFs contained motifs 1, 2, and 4, indicating that the structures of B3s are conserved (**Figure 3**, and **Supplementary Figures S1**, **2**). In the REM family, 44 B3 proteins had motifs 1 and 4, whereas the remaining 30 B3 proteins had only motif 1. Motif 25, which corresponded to the zinc finger CW domain (zf-CW), was found in nearly all members in the VAL subgroup. The ARF family is more conservative in terms of the number and distribution of motifs. According to domain analyses, we found that a number of the B3 proteins contained two B3-specific domains (B3 and B3_DNA), indicating specific characteristics of the B3s proteins. The domain analysis indicates that all 160 members belong to the B3 gene family. However, there were a number of variations in the domain number and types between the different B3 proteins. For example, in the ARF family, 24 proteins had three conserved domains (B3, Auxin_resp, and AUX_IAA superfamily), and 32 proteins had only two conserved domains (B3 and Auxin_resp) (**Supplementary Figure S3**). Among 10 VALs, 9 contained a zf-CW domain and a B3_DNA domain (**Figure 4A**). In the RAV family, 8 members (PagRAV2-A1/2, PagRAV2-B1, PagRAV3-B, PagTEM2-A1/2, and PagTEM2-B1/2) contained B3 and AP2 domains, whereas the rest of the members had only one B3 domain (**Figure 4B**). REM proteins showed more variations in domain number and types than the other family B3 proteins. For example, 31 REM members contained multiple B3 domains (**Supplementary Figure S4**). Gene structure analysis showed that 15% of B3 genes in *P. alba × P. glandulosa* contained only one exon, with incomplete untranslational region (UTR), and 3.8% had very long transcripts, potentially resulting from suboptimal genome annotation.

The promoter region among different B3 genes showed variable cis-acting elements. These cis-acting elements were involved in hormone metabolism, stress response, flavonoid biosynthesis, and so forth (**Figure 3**; **Supplementary Figures S1**, **2**), such as some hormone metabolism-related motifs, including TGA-element with the AACGAC binding site, TCA-element with the TCAGAAGAGG binding site, MeJA-responsiveness (methyl jasmonate) with the TGACG/CGTCA binding site, and ABRE element with the ACGTG binding site. Among them, ~25% (40 out of 160) B3 genes harbored TGA elements, ~58% (89 out of 160) contained TCA elements, ~70% (112 out of 160) contained MeJA elements, and ~75% (125 out of 160) contained ABRE elements. Cis-acting elements involved in auxin responsiveness (AuxRR-core) were found only in the promoter regions of the ARF (10 out of 56) and REM (7 out of 74) families (**Supplementary Figure S5**).

### 3.3 Expansion patterns of B3 genes in *P. alba × P. glandulosa*

Gene duplication events contribute to gene proliferation in the plant kingdom and often evolve into partition existing functions to form sub-functionalizations or neo-functionalizations (Airoldi and Davies, 2012; Lee & Irish, 2011). According to these criteria, we utilized the shinyCircos software to perform a genome-level collinearity analysis of the B3 genes (Yu et al., 2018). **Figure 2** (inside of the circle) showed the associated gene pairs of B3 genes. Out of 160 genes, 141 were discovered to possess paralogous genes, which suggests that B3 genes have undergone significant gene duplication occurrences. The B3 genes, which exist widely on 19 pairs of homoeologous chromosomes, are broadly distributed. Moreover, two or more genes found on the same chromosome within a 200 kb region were likely to be the result of tandem duplication (Houb, 2001). We observed that 44 B3 genes were clustered by 13 tandem duplication events. Among them, 7 chromosomes (Chr11A, Chr06A, Chr14A, Chr14B, Chr04B, Chr15A and Chr03B) had one duplication and 3 chromosomes (Chr4A, Chr15B, and Chr12B) had two duplications. It is surprising that the majority of genes belong to the REM family, suggesting that REM has undergone a greater degree of evolutionary change when compared to other B3 families. In addition to tandem duplication, many B3 genes resulted from segmental duplication events, such as PagVAL1-A1 and PagVAL1-A2 being located in Chr16A and Chr06A, respectively.

### 3.4 Expression patterns of B3 genes in three tissues of *P. alba × P. glandulosa*

To understand the expression patterns of these 160 B3 genes, we quantified their expression levels in three tissues, including leaf, xylem, and phloem, using the RNA-seq data from 6-month-old and 10-year-old trees. Four VAL subfamily genes, *PagVAL2-A1*, *PagVAL2-A2*, *PagVAL2-B1*, and *PagVAL2-B2*, were highly expressed in the differentiating xylem at two age trees, and the expression level increased with age, indicating that they may play important roles in xylem development. Two RAV family genes, *PagTEM2-B2* and *PagRAV4-A*, were also highly expressed in differentiating xylem of two age trees at the same level. The other 18 genes in the RAV family were preferentially expressed in leaves, such as *PagNGA2-B*, *PagNGA2-A2*, and *PagNGA1-A1*. In addition, many genes were not expressed in the 6-month-old trees but were highly expressed in 10-year-old trees, such as *PagVAL3-A2*, *PagARF2-B3*, and *PagREM2-A1* (**Supplementary Figures S6**–**8**).

### 3.5 Collinearity and evolutionary relationships of xylem formation-related B3 genes among seven species

**Figure 5:**
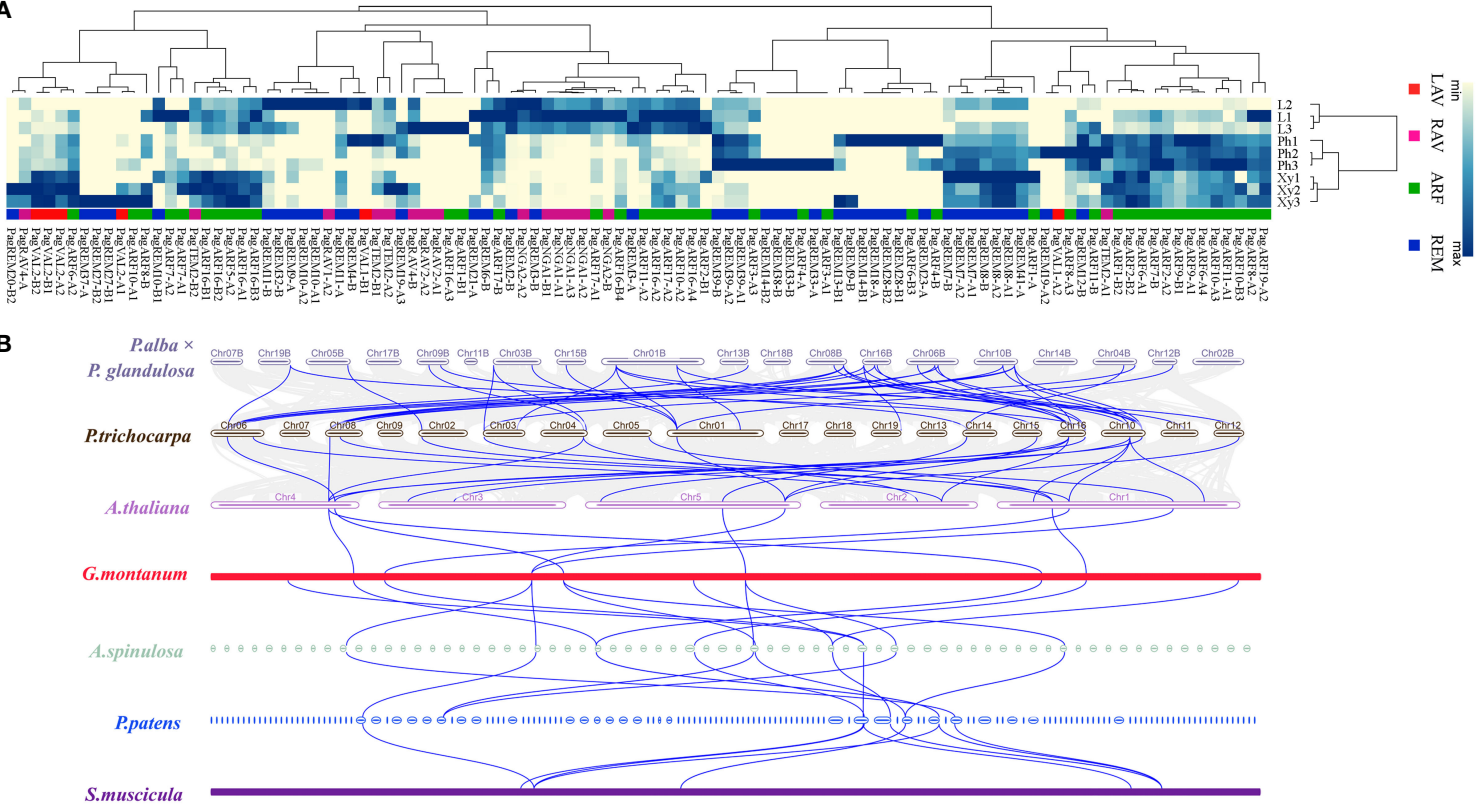
Expression analysis of B3 family genes in three tissues and collinearity analysis among different species. **(A)** A heatmap shows the expression level of 104 genes in different subfamilies (rows columns). L, leaf; Ph, phloem; Xy, xylem. **(B)** Multi-collinearity analysis of seven species. A blue line between two species means collinearity genes among different species.

Eukaryotic genomes vary in their level of synteny and collinearity, referring to the degree to which genes are retained on the same chromosomes and in the same order, respectively (Tang et al., 2008). To investigate the origin, evolutionary history, and potential functions of the *P. alba × P. glandulosa* B3 genes, we examined genomic synteny of *P. alba × P. glandulosa* with *A. thaliana* (herb), *P. trichocarpa* (woody), *G. montanum* (woody), *A. spinulosa* (fern), *P. patens* (moss) and *S. muscicola* (green algae). Based on expression pattern results, 18 genes that were highly expressed in the differentiating xylem of six-month-old trees were used to perform BLASTp with proteins in the above seven species to find their homologs (**Figure 5A**) (**Supplementary Table S3**). We obtained a total of 54 homologous proteins that corresponded to the 15 B3 proteins in *P. alba × P. glandulosa*. These 54 proteins included 8 in *P. patens*, 10 in *A. spinulosa*, 13 in *P. trichocarpa*, 9 in *Arabidopsis*, 9 in *G. montanum*, and 5 in *S. muscicola* (**Figure 5B**; **Supplementary Table S3**). We observed that most paralogous genes in *P. alba × P. glandulosa* corresponded to one gene in *Arabidopsis*: for example, *AT5G62000* was orthologous to *PagARF2-B2* and *PagARF2-B4*, and *AT1G59750* was orthologous to *PagARF1-B1* and *PagARF1-B2*, supporting the whole-genome duplication in trees (Tuskan et al., 2006). On the other hand, *AT5G62000* and *AT1G59750* corresponded to the same gene *TnS000811647t03* in *A. spinulosa*, indicating gene extraction in poplar. In all, our result suggests that they shared a common ancestor during evolution.

### 3.6 Gene co-expression analysis

**Figure 6:**
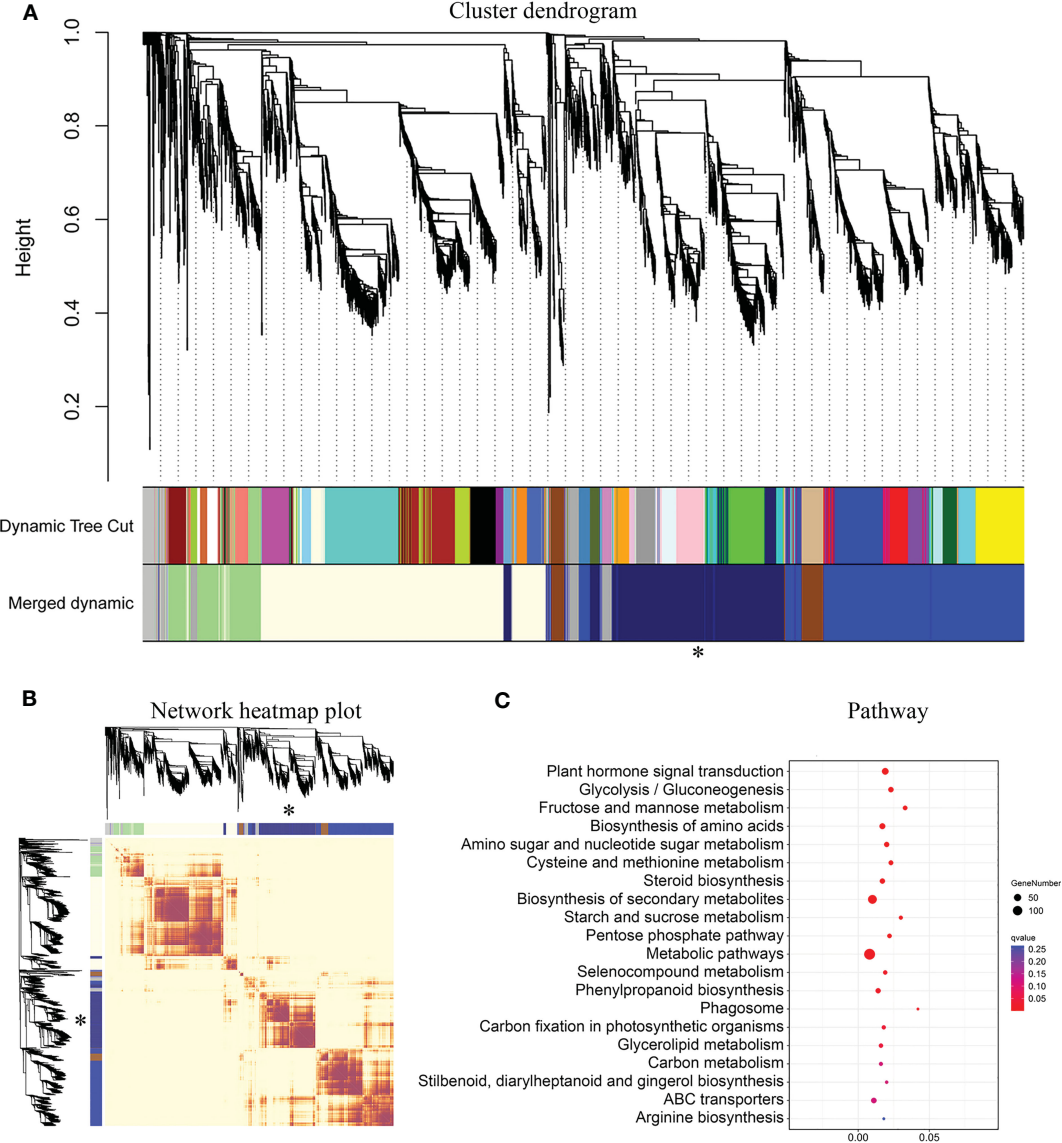
Result of WGCNA analysis and KEGG analysis of midnight-blue module. **(A)** Hierarchical clustering gene module in two different ages poplar. **(B)** Gene co-expression network heatmap of different modules. **(C)** KEGG analysis for the midnight-blue module. *, The key module related to wood formation. The different colors in A and B represent different modules. The heatmap describes adjacencies among genes in analysis. The dark color means a high correlation between two genes. Light-luminance color means a low correlation between two genes. The size of the pot means gene number, and darkblue means significantly enriched pathways.

Gene co-expression analysis can help identify genes with similar expression patterns. These genes may interact and co-regulate each other, exhibit similar functions, or be involved in the same signaling pathways or physiological processes. To further understand the correlation of these 160 B3 genes in wood formation, we conducted Weighted Correlation Network Analysis (WGCNA) using the RNA-Seq data of leaf, xylem, and phloem from 6-month-old and 10-year-old trees to construct a co-expression network centered on the 6 tissue-differentially expressed genes. As shown in **Figure 6A**, we obtained a total of 6 cluster dendrogram modules. Among them, the network in the same module has a strong correlation (**Figure 6B**). One of the modules centered on four B3 genes (*PagVAL2-A1*, *PagVAL2-B1*, *PagARF3-A1*, *PagARF3-B1*) is related to the wood formation (826 genes). KEGG enrichment analysis showed that these genes shared 20 significantly enriched pathways (**Figure 6C**). The shared pathways include biosynthesis secondary metabolites pathway, metabolic pathway, and phenylpropanoid biosynthetic pathway.

**Figure 7:**
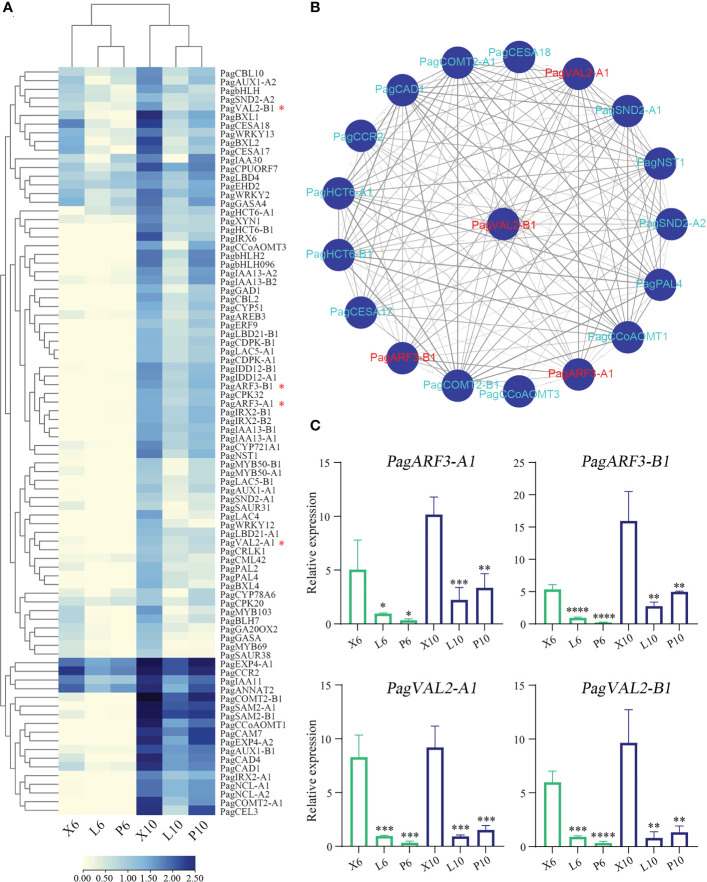
Gene expression profile in a wood formation-related module in poplar and co-expression analysis of B3 family genes. **(A)** Heatmap shows the expression of DEGs associated with wood formation. **(B)** Co-expression network of one tissue differentially expressed B3 gene *PagVAL2-B1*. **(C)** Four B3 family gene expression levels based on RT-qPCR. X6, L6, and P6 represent the xylem, leaf, and phloem of 6-month-old trees. X10, L10, and P10 represent the xylem, leaf, and phloem of 10-year-old trees. The relative expression levels are shown as the means ± SD of three biological replicates. The significance between xylem and phloem and between xylem and leaf was analyzed using Dunnett’s multiple comparisons test (*p ≤ 0.05, **p ≤ 0.01, ***p ≤ 0.001, ****p ≤ 0.001).

**Figure 8:**
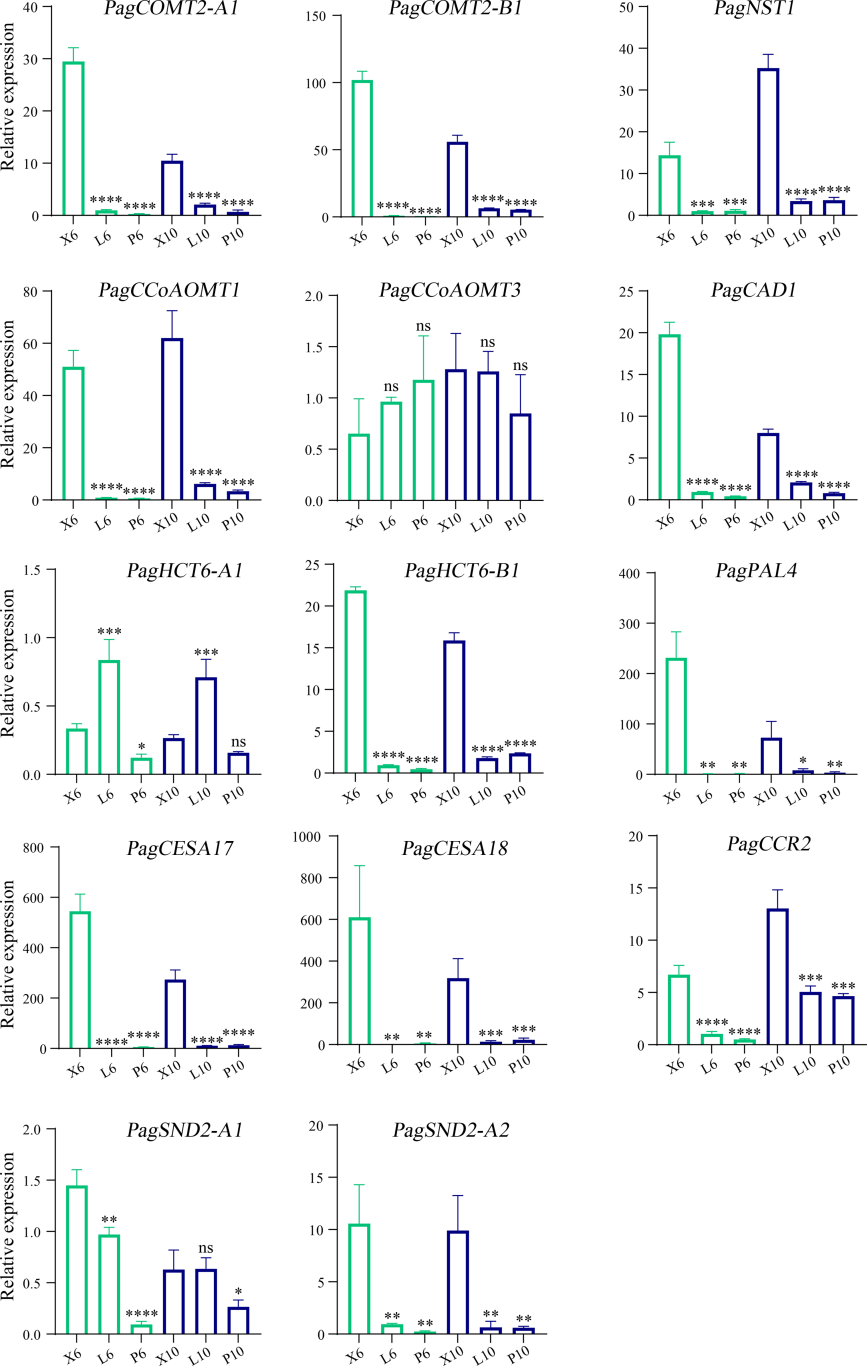
Gene expression levelsGene expression levels determined by RT-qPCR. X6, L6, and P6 denote the xylem, leaf, and phloem, respectively. The green and blue columns represent six-month-old and 10-year-old poplar trees. X6, L6, and P6 represent the xylem, leaf, and phloem of 6-month-old trees. X10, L10, and P10 represent the xylem, leaf, and phloem of 10-year-old trees. The relative expression levels are shown as the means ± SD of three biological replicates. The significance between xylem and phloem and between xylem and leaf was analyzed using Dunnett’s multiple comparisons test (*p ≤ 0.05, **p ≤ 0.01, ***p ≤ 0.001, ****p ≤ 0.001). ns, not significant.

To investigate the effects of B3 genes on the lignin biosynthetic pathway, we examined the expressions of the 86 genes involved in the secondary cell wall and phenylpropanoid biosynthetic pathways during wood formation. Among them, 36 genes were highly expressed in the differentiating xylem of 6-month-old trees, and most genes were highly expressed in the differentiating xylem of ten-year-old trees. There were fourteen genes co-expressed with these four B3 genes, such as lignin synthase genes *PagCOMT2*, *PagCAD1*, *PagCCR2*, *PagCAD1*, *PagCCoAOMT1*, and SCW TF genes *PagSND2* and *PagNST1* (**Figures 7A, B**). To further validate the results of RNA-Seq, we used RT-qPCR to quantify expression levels of the eighteen lignin biosynthetic pathway genes in leaf, phloem, and xylem of the two ages poplar. Among these 86 genes that were highly expressed in the xylem, 4 belonged to B3 genes, and these four genes (*PagVAL2-A1*, *PagVAL2-B1*, *PagARF3-A1*, and *PagARF3-B1*) were highly expressed in the differentiating xylem of two ages, indicating that they may play important roles in xylem development **Figure 7C**. Among the 14 genes that were co-expressed with the four B3 gene, 12 were highly expressed in the developing xylem of two ages trees **Figure 8**. Among the 12 genes, *PagNST1*, *PagCCoAOMT1*, and *PagCCR2* were significantly increased in 10-year-old tree compared to 6-month, while the remaining nine genes showed opposite expression trends.

## 4 Discussion

The plant-specific superfamily of B3 transcription factors is distinguished by the existence of one or more B3 domains, or a fusion of B3 domains with supplementary domains like AP2 (APETALA2), AUX_IAA, and zf-CW (Peng and Weselake, 2013). The B3 transcription factor superfamily, comprises four families, among which the LAV(LEC2/VP1/VAL) and ARF families are well-studied in *Arabidopsis*, characterized by diverse functions in plant growth and development, and extensively studied in *Arabidopsis* (Yamasaki et al., 2004; Swaminathan et al., 2008; Yamasaki et al., 2008; Agarwal et al., 2011). In contrast, the identification and characterization of B3 genes in woody plants is limited. New insight into this superfamily can be obtained through genome analysis. In this study, we performed a comprehensive characterization of B3 genes in *P. alba × P. glandulosa* and investigated their expression profiles in leaf, phloem, and xylem. We identified 160 putative B3 genes in the genome of *P. alba × P. glandulosa*, showing a discrepancy of gene number in each family with *Arabidopsis*. Different species are expected to have varying numbers of B3 genes in each family, and the number of B3 genes identified in different studies may also be influenced by the use of different database sources, methods, and parameters (Romanel et al., 2009; Peng and Weselake, 2013). For example, previous studies have classified B3 proteins lacking typical AP2 domains as members of the RAV family (Magnani et al., 2004; Kim et al., 2006). Similarly, in the LAV family, we identified only 10 VAL subgroup members, while LEC2-ABI3 was not identified according to our analysis; this could be due to the protein sequence variation from species to species. In addition to the B3 domain, the VAL members contain a zf-CW domain, with the one exception of PagVAL3-A1. For the RAV family, our results are consistent with previous results showing that some proteins contain the B3 and AP2 domains (Alvarez et al., 2009). Based on the evolutionary relationships between RAV proteins in *Arabidopsis* and *P. alba × P. glandulosa*, it was found that 8 out of 20 RAV proteins had AP2 domains. In the ARF family, 24 out of 56 members contained three domains (B3, Auxin_resp, and AUX_IAA), while the remaining 32 contained only two domains (B3 and Auxin_resp). The same observations have been reported in other plants, such as *Arabidopsis*, apple, and papaya (Li et al., 2015; Peng et al., 2020). We speculated that loss of the zf-CW, AP2, and AUX_IAA domains occurs during evolution. In a gene family, the emergence of new members can occur through domain duplication or loss. For example, in the MYB family, the plant-specific R2R3 organization is thought to have evolved from an R1R2R3-type ancestral gene from which the first repeat domain R1 motif was lost (Braun and Grotewold, 1999; Riechmann et al., 2000; Dias et al., 2016).

The domain arrangement observed in REM proteins is more intricate compared to other families. Thirty-one members of the REM family contain multiple B3 domains: for example, PagREM6-A/B contained the highest number of five (**Supplementary Figure S4**). The complexity of domain architecture in REM proteins suggests that they may have evolved from one or more domain duplication events, which can lead to functional diversification over the course of evolution. However, the study of genes with domain duplications is often challenging as the functional redundancy of many proteins can make it difficult to ascertain their specific roles (Waltner et al., 2005). Previously, domain duplication has been reported in the RAV family as well as other families of transcription factors. For example, in the liverwort *Marchantia polymorpha*, a RAV protein has been identified to contain two B3 domains, indicating the occurrence of domain duplication in this protein family (Swaminathan et al., 2008). Among the 167 basic helix-loop-helix (bHLH) proteins that have been identified in the rice genome, it has been discovered that one particular protein (OC_Os01g09930) contains two bHLH domains that are duplicated (Li et al., 2006).

Gene duplication is the primary source of the evolution of genes and gene families (Cannon et al., 2004). Previous research has demonstrated that the expansion of the B3 superfamily in *Brassica rapa* is primarily driven by tandem duplication, as evidenced by the presence of both two-copy and multi-copy tandem-arrayed B3 genes in its genome (Peng and Weselake, 2013). Duplicated B3 genes and other transcription-factor genes have also been found in the *Arabidopsis* genome (Riechmann et al., 2000; Romanel et al., 2009). Similar findings have been reported in *Cicer arietinum*, where *CaARF4/CaARF5* and *CaARF21/CaARF22* are duplicated in tandem but exhibit distinct structural differences (Die et al., 2018). Likewise, the REM family we identified is distributed on many chromosomes (Chr04A/B, Chr09A/B, Chr14A/B, and Chr15A/B). Tandem duplications appear to have occurred in 40 genes among 74 members of the REM family. In addition, only 44 of all the 160 B3 genes are products of tandem duplication events. Consistent with this, we found that the rest of the gene belongs to segmental duplication. This analysis suggests that segmental duplication may happen more frequently than tandem duplication in *P. alba × P. glandulosa*, and that numerous duplicated chromosomal blocks are retained in their genome after duplications (Cannon et al., 2004).

The B3 domain is not specific for angiosperms. It is also present in genes from gymnosperms, ferns, mosses, liverworts, and green algae (Marella et al., 2006). Evolution and genomic synteny can provide clues to gene function, and can be an effective tools for gene family analysis in the species with available whole-genome sequences (Lyons et al., 2008; Zhang et al., 2012). There is only a single B3 gene in *Chlamydomonas reinhartii*, a single-celled green algae with available complete genome sequence, and an ortholog of this gene has been identified in green algaes *Mesostigma viride* and *S. muscicola*, suggesting that the B3 domain arose before the development of multicellularity in the plant lineage (Merchant et al., 2007). This single B3 gene in *C. reinhartii* is similar in structure to the existing VAL subgroup in the most recent common ancestor of green algae and higher plants. Thus it is hypothesized that the *C. reinhartii* B3 gene is the ortholog of the VAL subgroup (Swaminathan et al., 2008). In the metaphyte lineage, a series of gene duplication events occurred before the speciation event between mosses and vascular plants. The first led to the LEC2-ABI3 subgroup, and subsequent gene duplications led to the RAV, ARF, and REM families (Swaminathan et al., 2008). Because of the evolutionary diversity and differences in genome polyploidy between different species from green algae to vascular plants, we selected seven species for collinearity analysis using orthologous genes. Eighteen B3 TF genes that were highly expressed in the xylem were selected for collinearity analysis. These genes can be found to be homologous in seven species and have collinearity on chromosomes, suggesting that these B3 genes were already present in early subaerial/terrestrial plants (Wang et al., 2020). For example, *SM000256S08685* gene in *S. muscicola*, *PAC:32923104* gene in *P. patens*, *Aspi01Gene22980.t1* gene in *A. spinulosa*, and *AT4G32010.1* gene in *A. thaliana* are orthologs of *Pag.B16G000101* (*PagVAL2-B1*) gene in *P. alba × P. glandulosa*, belonging to the VAL subgroup. These findings suggest that the VAL genes have undergone a complex evolutionary history.

The B3 domain is highly conserved in B3 TFs, and it consists of 2 α-helices and 7 β-strands. Differences in amino acid residues may lead to different DNA binding ability or binding sequences of B3 proteins (Yamasaki et al., 2004; Waltner et al., 2005). The B3 domain of *Arabidopsis* VAL protein can recognize and bind to Sph/RY elements (CATGCA), where histone H3 lysine 27 trimethylation (H3K27me3) is marked and suppresses the transcription of downstream genes. When the B3 domain in VAL protein is destroyed, the expression of a downstream gene *DOG1* is significantly upregulated, indicating that VAL proteins function through B3 domain-mediated binding (Chen et al., 2020). In addition, The AP2 and B3 domains in RAV1 protein can bind to the CAACA and CACCTG motifs, respectively, promoting the high-affinity activity of RAV1 in binding DNA sequences (Kagaya et al., 1999). In the ARF protein, the DNA-binding domain (B3 domain and Auxin-resp domain) is in the N-terminal region, while the protein-protein interaction domain (AUX_IAAs) is in the C-terminal portion (Wang et al., 2007). The promoter regions of B3 genes contain hormone-responsive elements such as ABA response elements (ABREs) and GA response elements (TCA motif and GARE sequences), and consistent some B3 TF genes are induced by hormone. The study on the ABA-dependent activation of the *CRC* (*CRUCIFERIN C*) promoter by the LEC1-NF-YC2 trimer indicates that ABRE motif is a necessary cis-element. Moreover, a seed-specific ABRE-binding protein, bZIP67, can functionally interact with LEC1-NF-YC2 to enhance the activation, while bZIP67 alone cannot activate the promoter. These results indicate that ABRE motif plays an important role in B3 protein-mediated regulation. (Curaba et al., 2004; Gazzarrini et al., 2004; Yamamoto et al., 2009).

The patterns of gene expression can serve as a critical indicator of the function of genes. In this study, some B3 genes exhibit tissue-specific expression patterns in *P. alba × P. glandulosa*: for example, *PagARF10-A2*, *PagARF16-A2*, *PagARF17-A1*, and *PagARF11-A2* were found preferentially expressed in leaves, and *PagVAL2-A1/A2/B1/B2* genes were preferentially expressed in xylems. In addition, 31 ARF genes were found to be expressed in all three tissues, among which nine were preferentially expressed in the differentiating xylem. Moreover, it seems that many ARFs function redundantly, thus single loss-of-function mutants do not exhibit growth and developmental phenotypes. In *Arabidopsis*, the *arf7 arf19* double mutant has a strong auxin-related phenotype, including severely impaired lateral root formation and abnormal gravitropism in both hypocotyls and roots, which are not observed in the *arf7* and *arf19* single mutants (Okushima et al., 2005).

The VAL family genes *PagVAL2-B1* and *PagVAL2-A1* were highly expressed in the differentiating xylem of two age trees. Its specific expression in the xylem is consistent with our previous identification that it is a putative upstream of *PagCAld5H2*. Although studies have shown the essential role of VAL genes in regulating the transition from seed maturation to seedling growth, few of them have been studied for their functions in the process of tree development. As one of the most environmentally cost-effective and renewable sources, poplar wood has been widely used for timbering, paper making, and many other commercial applications (Wang et al., 2013). In trees, the secondary growth of stem and the lignin biosynthesis gives rise to wood formation (Zheng et al., 2021; Luo and Li, 2022). To understand their function, we investigated the co-expression gene network analysis, focusing on the one key module related to wood formation we identified, followed by pathways enrichment analysis. Our results from pathways analyses indicated that the differentially expressed genes (DEGs) across tissues and their corresponding network genes are enriched in many different pathways. Among which, *PagVAL2-B1* and that corresponding network genes are enriched in regulation of SCWs biosynthesis and phenylpropanoid biosynthesis. This result suggests that *PagVAL2-B1* can positively regulate secondary growth and lignin biosynthesis in poplar.The members belonging to the plant-specific B3-domain transcription factor family exhibit significant and diverse roles, particularly concerning vegetative and reproductive growth. We performed a detailed genome-wide analysis of 160 B3 family members based on the HMMER search and BLASTP method in *P. alba × P. glandulosa.* Motif, domain, promoter, and gene structure analyses were conducted for further identification. Gene duplication events and evolutionary analyses of B3 members were performed to illustrate the relationship within the *P. alba × P. glandulosa* and seven species genomes. Among the four B3 genes that were highly expressed in mature xylem, one (PagVAL2-B1), was studied for its co-expression relationships with other wood formation-related genes by WGCNA. Our analysis suggests that PagVAL2 family genes are involved in wood formation. Further using genetic transformation to study their specific roles in wood formation could help in the design to improve wood properties. Among the 160 B3 TF genes, 15% had incomplete coding region due to the annotation quality, and this may affect the gene structure analysis. The annotation needs further improvement to help the design in genetic transformation and CRISPR-based gene editing.

## Data availability statement

The original contributions presented in the study are included in the article/**Supplementary Material**. Further inquiries can be directed to the corresponding authors.

## Author contributions

MW, HL, and QL conceived the project. MW, HL, and QW performed the experiments. MW, HL, RL, and LY ran the program and visualized the data. MW wrote the draft, HL and QL edited the paper. All authors contributed to the article and approved the submitted version.
