# Supplementary material for: Genome-wide identification and expression profiling of B3 transcription factor genes in Populus alba × Populus glandulosa
Source: Front Plant Sci. 2023 May 30;14:1193065. doi: 10.3389/fpls.2023.1193065 (PMC10262750; doi:10.3389/fpls.2023.1193065)
Supplement: Supplementary file 1 [file DataSheet_1.zip › Supplementary_Materials/Supplementary Figures.docx]

Supplementary Material

Genome-wide identification and expression profiling of B3 transcription factor genes in *Populus alba × Populus glandulosa*

**Mingke Wei^1^, Hui Li^2^, Qiao Wang^3^, Rui Liu^1^, Linxi Yang^1^, Quanzi Li^1*^**

*** Correspondence:** Quanzi Li: liqz@caf.ac.cn

# Supplementary Figures and Tables

## Supplementary Figures


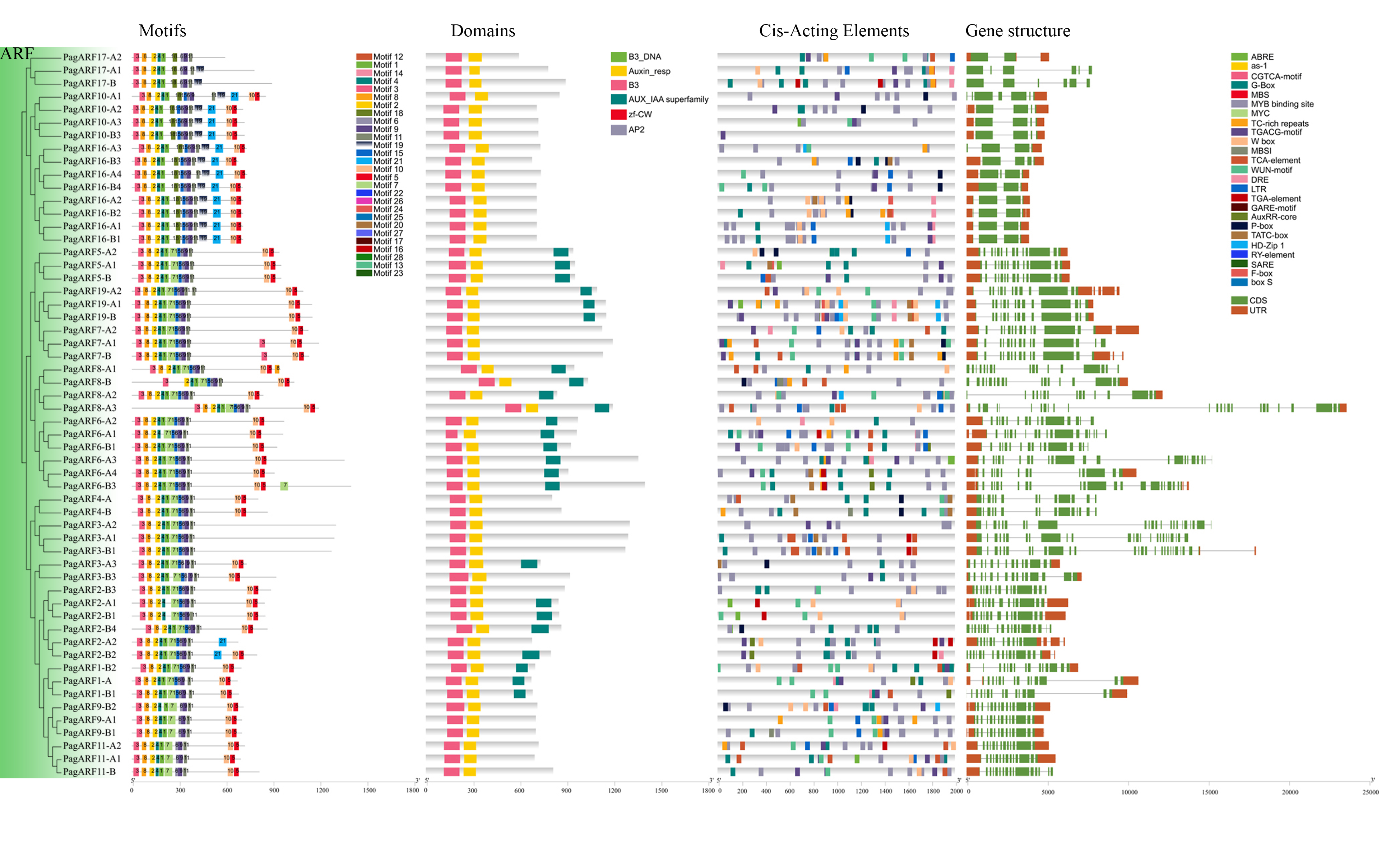


**Supplementary Figure S1.** Motifs, domains, promoters, and gene structures of the ARF families.


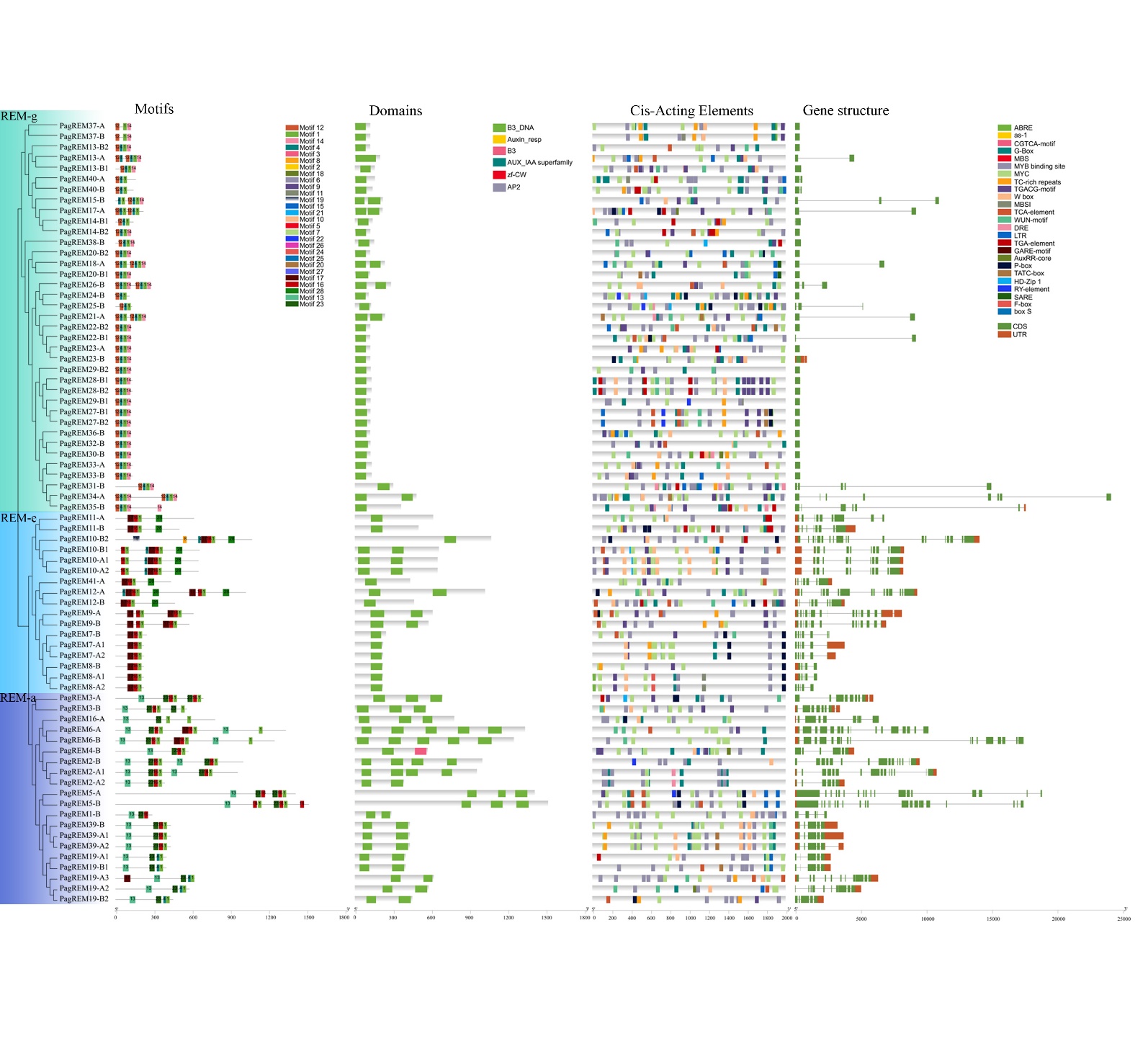


**Supplementary Figure S2.** Motifs, domains, promoters, and gene structures of the REM families.


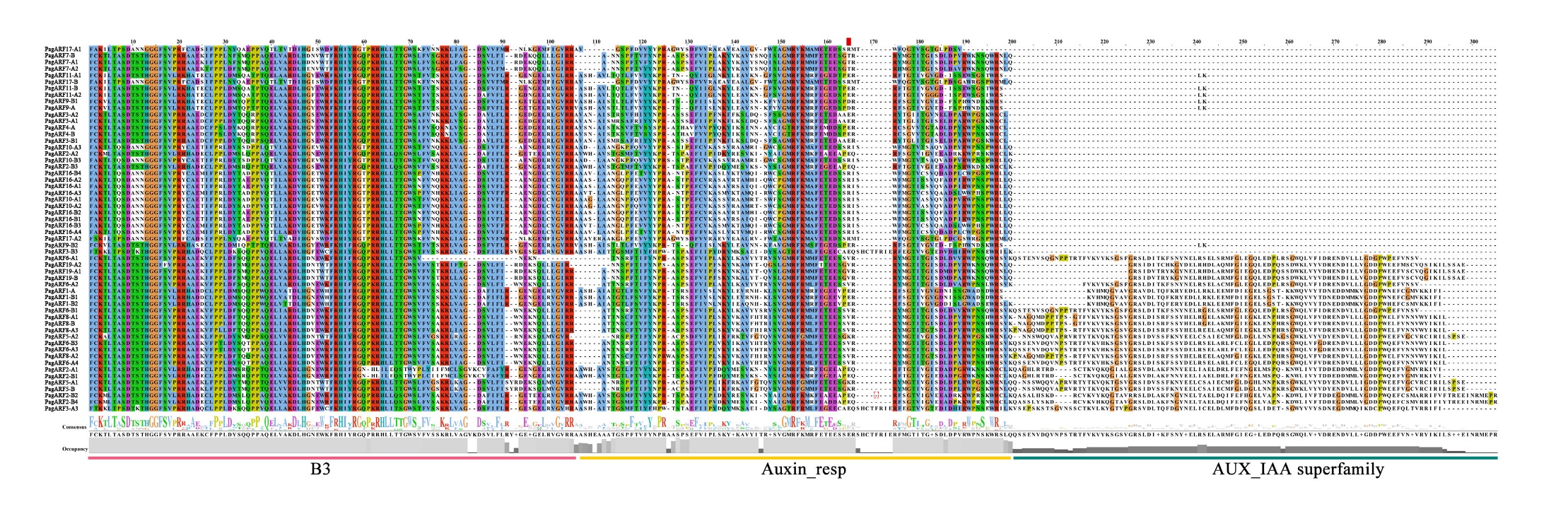


**Supplementary Figure S3.** Sequence alignment and seqlogo of ARF gene families.


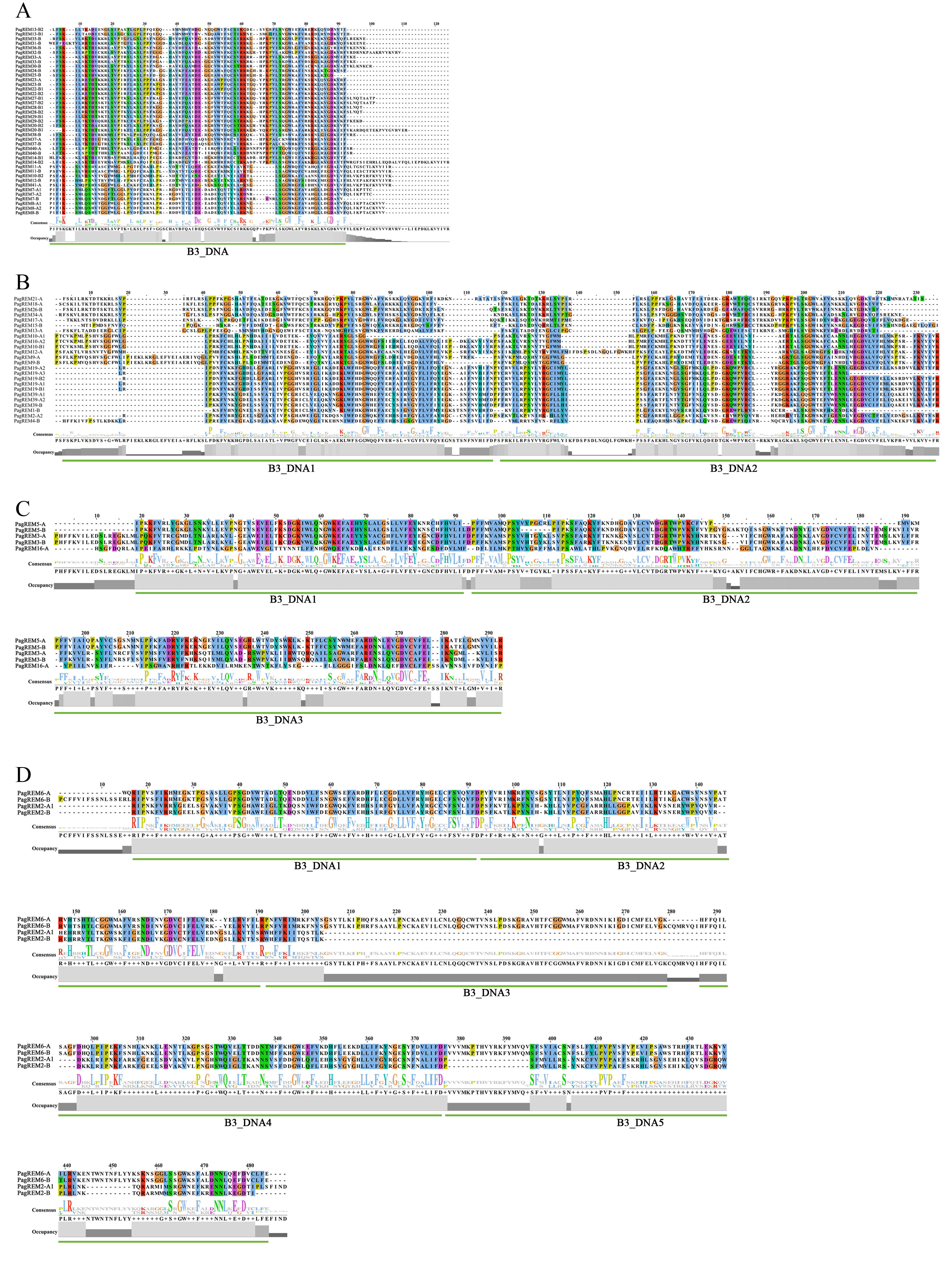


**Supplementary Figure S4.** Sequence alignment and seqlogo of REM gene families. These multiple B3 domains were classified into four groups, A, B, C, and D, which contained 1, 2, 3, 4, and 5 domains, respectively.


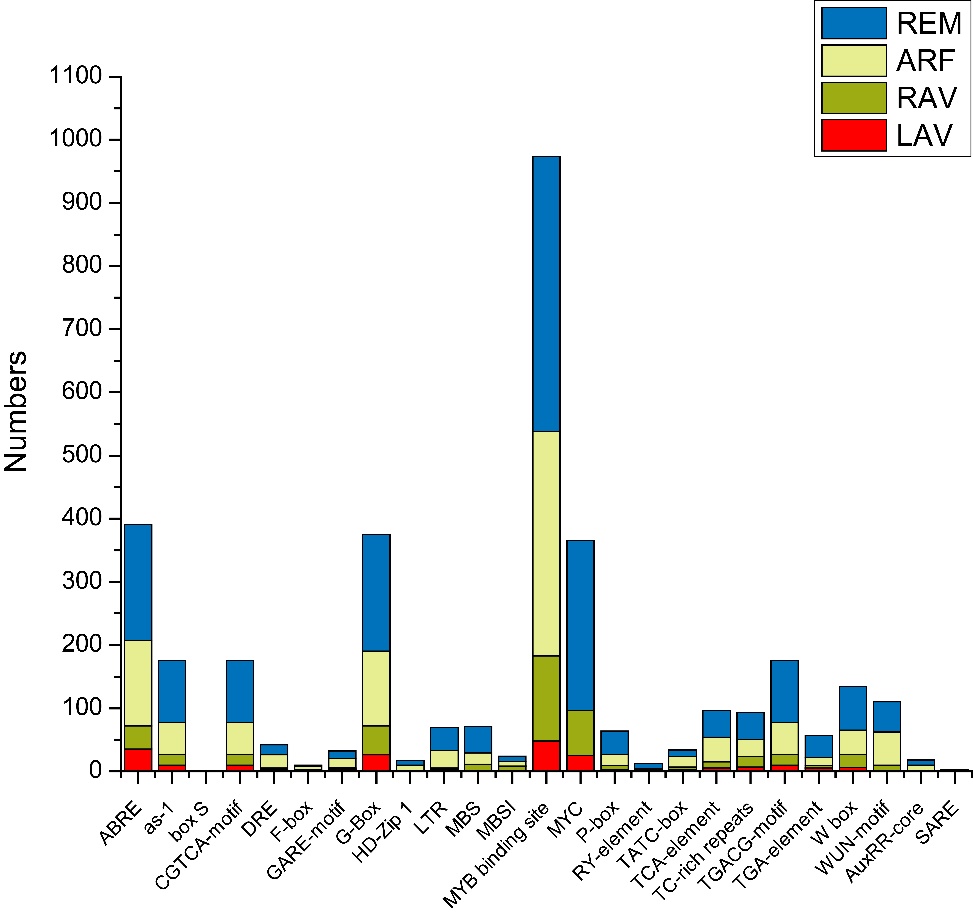


**Supplementary Figure S5.** Statistics of different cis-acting elements in the promoter of the identified 160 B3 genes. The 2 kb sequences of 160 B3 genes were analyzed with the Plant CARE program.


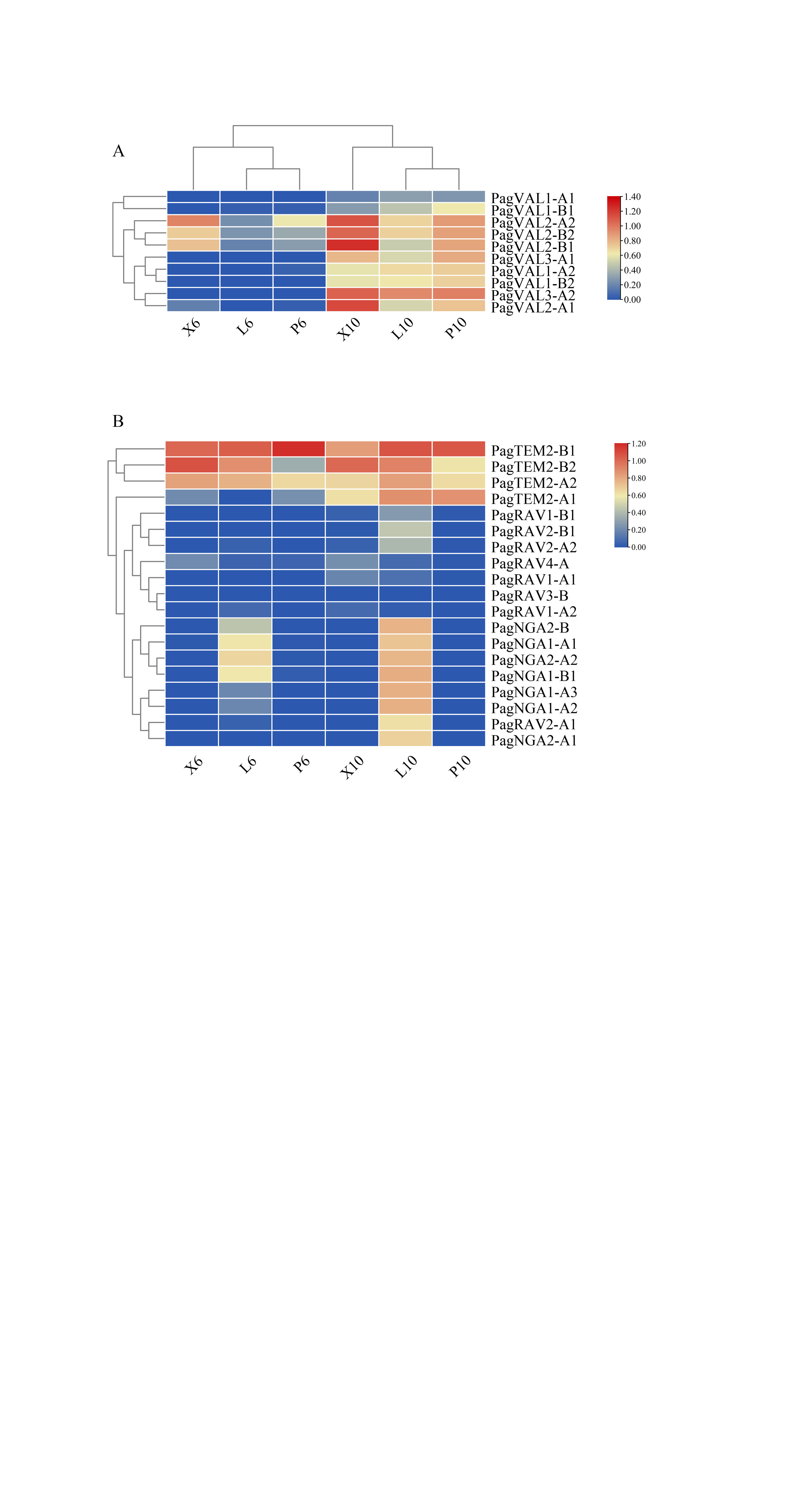


**Supplementary Figure S6.** Expression profile of the B3 family genes in two ages of poplar. (A) VAL (B) RAV. L6, P6, and X6 represent leaf, phloem, and xylem of 6-month-old trees. L10, P10, and X10 represent leaf, phloem, and xylem of 10-year-old trees.


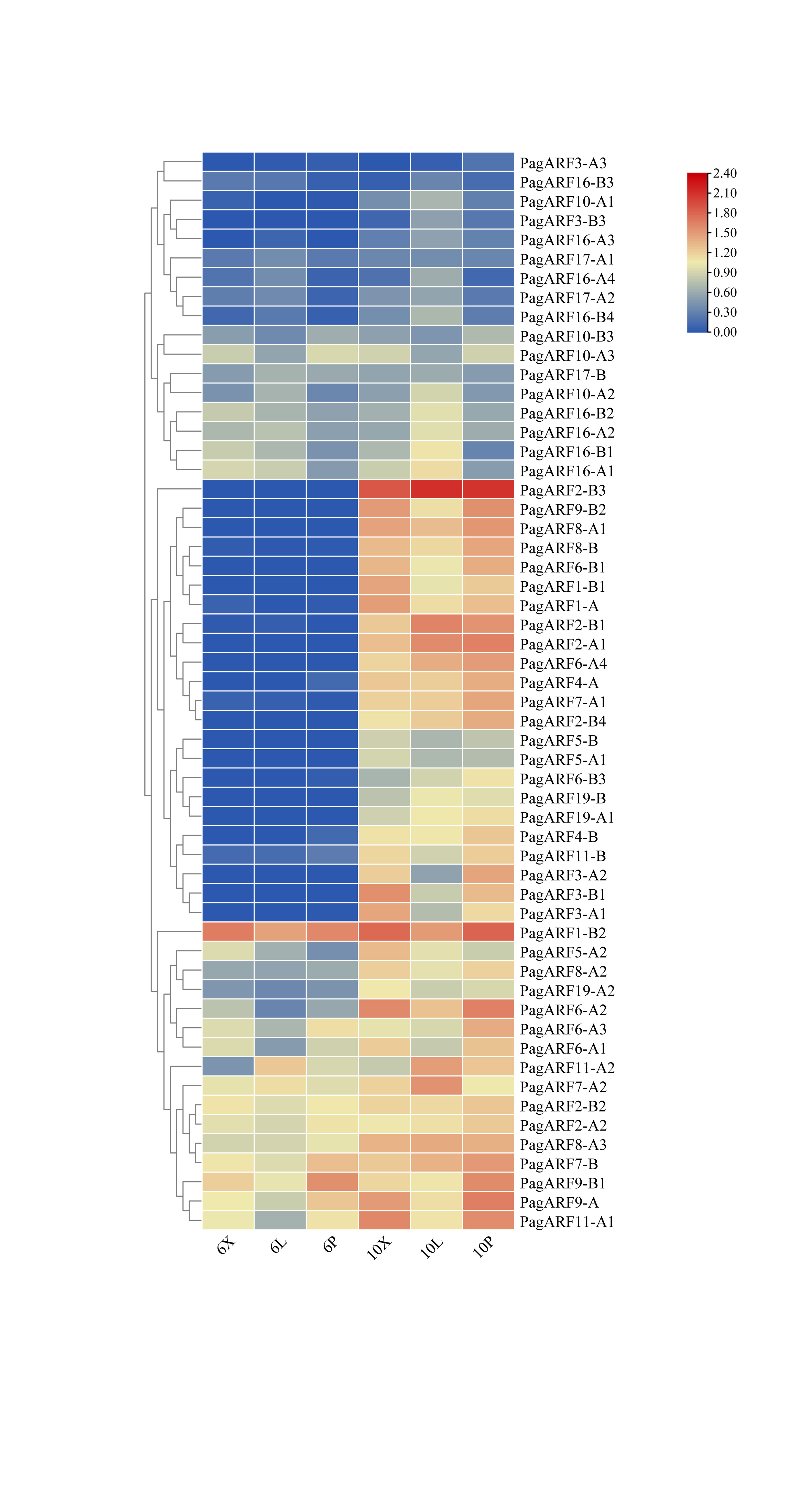


**Supplementary Figure S7.** Expression profile of the ARF family genes in two ages of poplar. L6, P6, and X6 represent leaf, phloem, and xylem of 6-month-old trees. L10, P10, and X10 represent leaf, phloem, and xylem of 10-year-old trees.

**
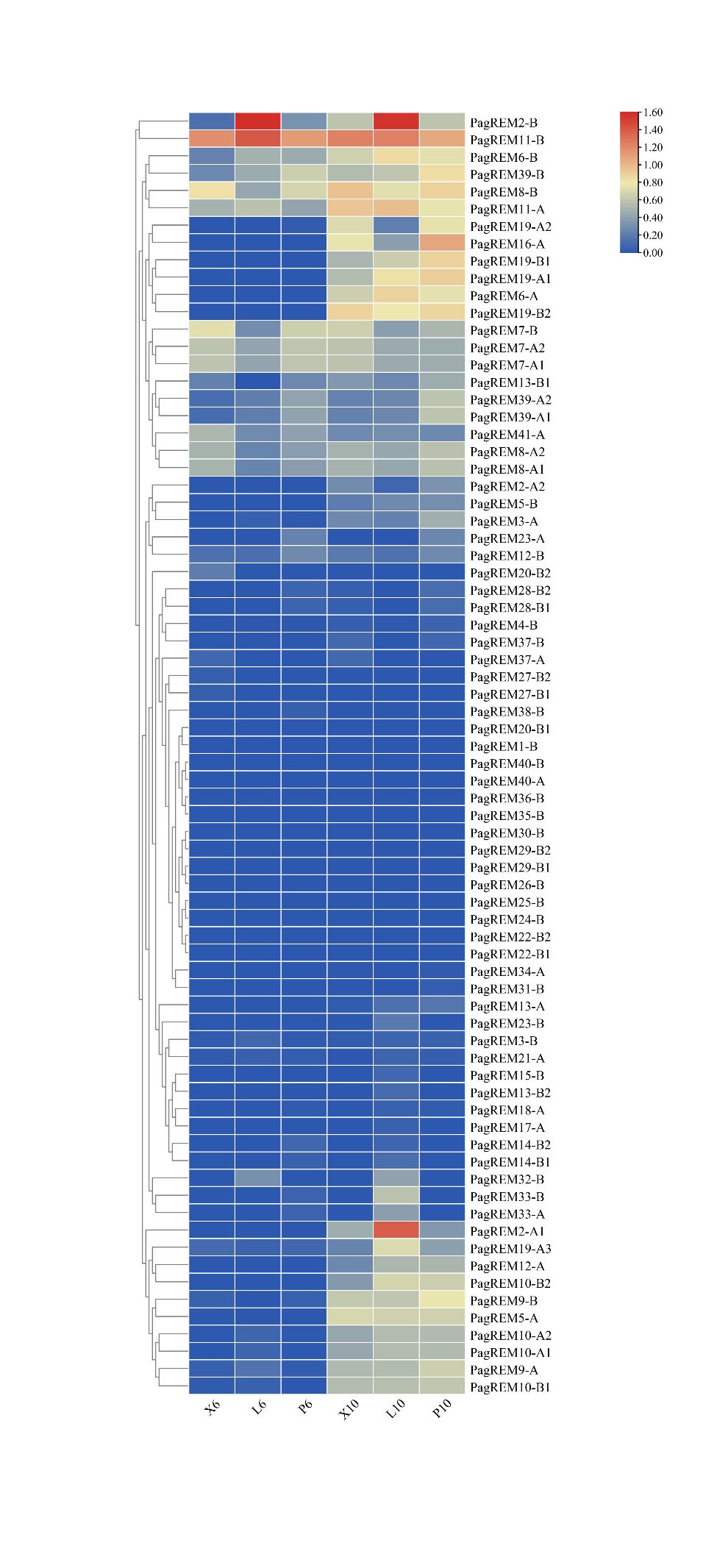
**

**Supplementary Figure S8.** Expression profile of the REM family genes in two ages of poplar. L6, P6, and X6 represent leaf, phloem, and xylem of 6-month-old trees. L10, P10, and X10 represent leaf, phloem, and xylem of 10-year-old trees.

**
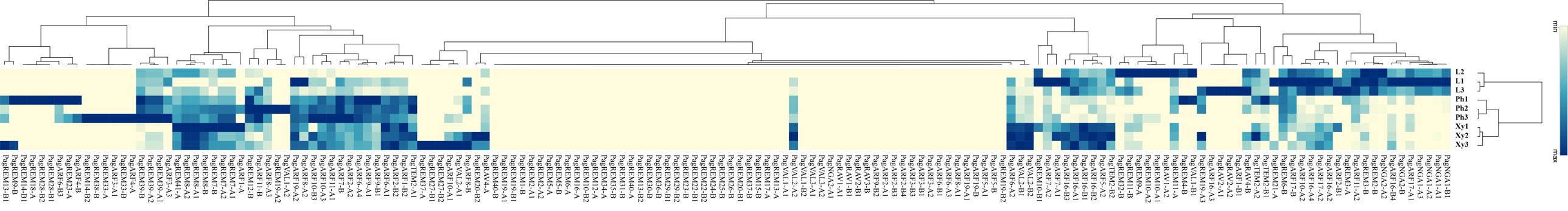
**

**Supplementary Figure S9.** Expression analysis of 160 B3 family genes in three tissues of 6-month-old tree. L: leaf; Ph: phloem; Xy: xylem.
